# Maternal Health and Green Spaces in China: A Longitudinal Analysis of MMR Based on Spatial Panel Model

**DOI:** 10.3390/healthcare7040154

**Published:** 2019-12-02

**Authors:** Ping Jin, Yushu Gao, Lingbo Liu, Zhenghong Peng, Hao Wu

**Affiliations:** 1 Department of Graphics and Digital Technology, School of Urban Design, Wuhan University, Wuhan 430072, China; jinping@whu.edu.cn (P.J.); gaoyushu@whu.edu.cn (Y.G.); pengzhenghong@whu.edu.cn (Z.P.); wh79@whu.edu.cn (H.W.); 2 Department of Urban Planning, School of Urban Design, Wuhan University, Wuhan 430072, China

**Keywords:** maternal health, public green space, green space coverage, spatial panel model, maternal mortality ratio

## Abstract

The positive impact of green spaces on public health has attracted increasing attention, and maternal health has also been shown to be related to green spaces. However, there are different kinds of green space indicators that represent different mechanisms for mitigating maternal health, and few studies have investigated the different relevance amongst them with longitudinal data. This study explores the correlation between various green space indicators and maternal health using spatial panel models with provincial data from China from 2007 to 2016. The results indicate that all kinds of green spaces could decrease maternal mortality, wherein public green spaces may play a key role. In terms of spatial correlation, an increase in green space coverage in adjacent provinces may also result in a slight decline in maternal mortality. This paper provides valuable insight into the correlation between maternal health and green spaces.

## 1. Introduction

Maternal health has been a key area of concern for public health and plays an important role in the Sustainable Development Goals (SDGs) of the World Health Organization (WHO) [1]. Despite the slow decline in the maternal mortality ratio (MMR) in recent years, there are still vast inequalities worldwide, which are more serious in developing countries [2,3]. China’s MMR has decreased rapidly, but there are still inequalities between rural and urban areas, inland and coastal regions [4], and even in recent years, substantial heterogeneity has also been detected at the county level [5]. The MMR of economically disadvantaged areas in the western regions is still high [6]. Although the mortality ratio of obstetric hemorrhage in China has been greatly reduced due to the enhancement of healthcare [7], the inequalities in maternal health still need to be paid attention to [8].

**Figure 1:**
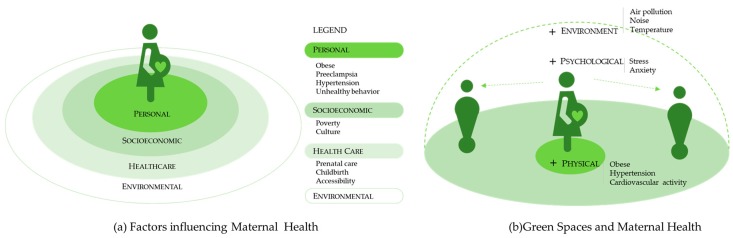
Maternal health and Green Spaces. (**a**) Factors influencing maternal health; (**b**) Green spaces and maternal health.

More and more studies have suggested that in the context of urbanization, the inequality of MMR could be related to the disparity in green space exposure during pregnancy, in terms of social deprivation [9] and environmental justice [10]. Previous studies have revealed four factors that may affect the inequalities of maternal health: personal state [11], socioeconomic status, healthcare condition [12], and environmental exposure [13] (Figure 1a), wherein green spaces play an important role in environmental intervention. Green spaces could benefit public health by increasing residents’ physical activities [14], relieving mental stress and depression [15,16], reducing noise and air pollution [17,18], and regulating high-temperature environments [19,20]. This mechanism may affect maternal health from physical, psychological, and environmental aspects as well (Figure 1b). For example, it has been proposed that appropriate physical exercise stimulated by public green spaces is beneficial to the mental and physical health of pregnant women, thereby reducing negative pregnancy outcomes [21,22,23,24,25,26,27,28]. Moreover, adverse pregnancy outcomes have been also connected with environmental factors, such as poor air quality [29,30] and high-temperature environments [31], which could be adjusted by green spaces.

However, it is still not known which kinds of green spaces would impose the crucial influence on maternal health, such as parks, forests, wetlands, and grasslands, in both public and private spaces, which are different not only in scale, function, and accessibility but also in vegetation coverage, ecological scale, and environmental quality as well [32]. Moreover, the substantial connection between green spaces and maternal health has seldom been observed [33], which has led to ambiguity in maternal health policymaking.

Previous studies were based primarily on cross-sectional datasets from relatively small regions, taking the Normalized Difference Vegetation Index (NDVI) as a measurement of green space and calculating vegetation levels within buffers between 50 to 1250 m around a residential address or the distance to a major green space [34,35]. Such experiments may ignore the influence of green spaces around the cities, as pregnant women may travel around, or even outside the cities, experiencing long-term exposure to green spaces, and the related variables of nearby cities may also affect maternal health. On the other hand, NDVI, as a single general green coverage indicator, may not reflect the distinguishing features of green spaces, as some specific kinds of green spaces could play a key role in maternal health.

This paper argues that based on longitudinal data and spatial autocorrelation analysis, additional variables of different kinds of green spaces at a macro-level might detect the association between green space and maternal health. Data for this study were provincial panel data from 2007 to 2016 in China, including indicators of maternal health, economy, healthcare level, urbanization rate, and green space. MMR was chosen as the independent variable, and the explanatory variables contain socioeconomic indicators and three kinds of green spaces, area of parks, area of green space, and the green coverage ratio of the built-up area, which may represent the quantity of public green spaces, general green spaces, and green space coverage, respectively. Based on quantitative analysis, this paper evaluated the spatial agglomeration effect of maternal health with Global Moran’s I, investigated the correlation between green spaces and maternal health by panel data model (PDM), and further explored the spatial correlation effect by spatial panel model (SPM).

This study should help to better understand the connection between green spaces and maternal health, and provide new sights into healthy city planning.

## 2. Materials and Methods

### 2.1. Variables and Data Source

This paper includes analyzed panel data from 31 provincial units in mainland China from 2007 to 2016, including 22 provinces, 5 autonomous regions, and 4 centrally-administered municipalities. For shorthand, this paper uses the term “provinces” for all types of provincial administrative regions in mainland China. The data on maternal health were extracted from the China Health Statistics Yearbook, 2007–2016 [36], and the data on green spaces and other socioeconomic data were acquired from the China Statistics Yearbook, 2007–2016 [37].

**Figure 2:**
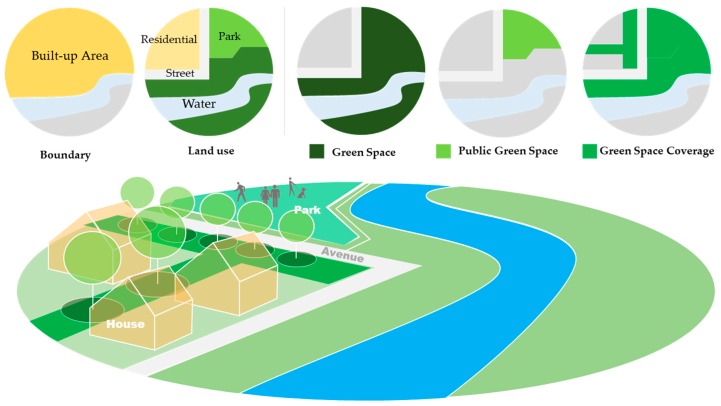
Definition of public green space (GS), GS coverage, and GS.

(1) Maternal health indicators used as dependent variables included: maternal mortality ratio (MMR) and high-risk parturient ratio (HPR), which were selected as two typical indicators and dependent variables for maternal health. High-risk parturient refers to mothers who have certain pathological factors during pregnancy, which may endanger pregnant women, fetuses, and newborns, and lead to dystocia. The number of live births refers to newborns who have reached the age of 28 weeks or more in the year and have the four vital signs of heartbeats, breathing, umbilical cord fluctuation, and voluntary muscle contraction after delivery. HPR was the proportion of high-risk parturient and live births. 

(2) Green space indicators used as independent variables included: three statistic indices in the China Statistic Yearbook, i.e., green space, area of parks, area green space, and the green coverage ratio of built-up areas, were chose to represent variables of public green spaces (public GS), green space coverage (GS coverage), and green space (GS), whose spatial boundaries follow in an ascending order [38]. Public GS refers to the green spaces which are open to the public, focusing on physical activities. It also has some recreation and service facilities. GS coverage is the vertical projection area of all green plants in the urban built-up area, which contains not only public green space, but also residential green areas, avenue trees, and where applicable, trees canopy cover. Green spaces, which included public green spaces, productive plantation, environmental protection, and unoccupied wildlands inside the administrative boundary, has the largest area among them (Figure 2).

(3) Supplementary independent variables used relating to socioeconomic and healthcare level included: other factors that affect maternal health, such as economic level and medical services [39,40,41,42,43], etc., prompted this study to also include the flowing control variables, the provincial indices of GDP per capita (10,000 yuan), the number of medical workers (10,000 persons, MediWorker), population density (10,000 persons/100 km^2^, PopDen), the number of hospital beds (10,000 beds, MediBed), and urbanization rate (urban R) were also chosen as control variables. The contents in parentheses are the short name and the corresponding unit of each variable.

In order to avoid heteroscedasticity [41], a logarithmical transformation was applied to all the variables except MMR, HPR, urban R, and GS coverage ratio, which have the unit of percentage.

### 2.2. Methods

**Figure 3:**
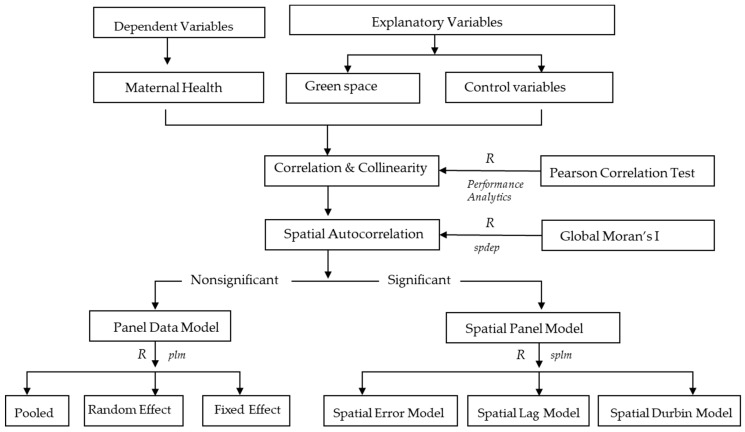
The workflow of methodology.

As a spatial correlation would be explored with the longitudinal data, the spatial panel model was preferred, based on a test of Pearson correlation and spatial autocorrelation. The entire workflow with the open source software R and corresponding packages is presented in Figure 3.

(1) Global Moran’s I

Global Moran’s I is used to identify whether there is spatial autocorrelation in the spatial distribution of MMR and other explanatory variables. The value of Moran’s I, ranging from 1 to −1, indicates the changing state from extremely spatially clustered to spatially dispersed. A larger positive Moran’s I value means stronger spatial correlation, and smaller negative value means greater spatial disparities. When the value of Moran’s I approaches zero, the geographic factor shows a random distribution with no spatial autocorrelation.

Moran’s I is defined as:

(1) $$I=\frac{n}{W}\frac{\sum_{i=1}^{n}\sum_{j=1}^{n}w_{ij}(x_{i}-\bar{x})}{\sum_{i=1}^{n}{\left(x_{i}-\bar{x}\right)}^{2}}\;\left(i\neq j\right)$$

(2) $$W=\sum_{i=1}^{n}\sum_{j=1}^{n}w_{ij}\;\left(i\neq j\right)$$

*x* is an independent variable with its corresponding mean value $\bar{x}$. $w_{ij}$ is the spatial weight between geographic locations *i* and *j*, and *n* is the total number of locations. The sum of all spatial weights $w_{ij}$ is *W*.

(2) Panel Data Model

Based on longitudinal data, a panel data model (PDM) is capable of describing individual differences and overcoming the problem of multicollinearity, providing more information and higher estimation efficiency [44]. A general PDM can be expressed as:

(3) $$y_{it}=\alpha_{it}+\mu_{it}+\Sigma \beta_{it}x_{it}$$

Wherein, *y* and *x* are the dependent and explanatory variables, respectively, *i* and *t* are labels for various observed individuals and times, *α* is the intercept, *β* is the coefficient, and *μ* is a random perturbation term. A PDM often contains three sub-models: a pooled OLS model, a random effect model, and a fixed effect model.

(3) Spatial Panel Model

A spatial panel model (SPM) can test the spatial correlation of panel data variables. The establishment of the spatial weight is key to the spatial panel model, and it is also the embodiment of the regional spatial impact. The weight matrix is often defined as a binary variable based on the Rook adjacency rule. To consider the spatial correlation, the spatial weight $w_{ij}$ is added to Equation (3), thus a general SPM can be written as:

(4) $$y_{it}=\alpha_{it}+\rho \sum w_{ij}y_{it}+\delta \sum w_{ij}x_{it}+\gamma_{t}+u_{it}$$

(5) $$u_{it}=\lambda \times \sum w_{ij}\times \mu_{it}+\varepsilon_{it}$$

wherein *μ* is a random error term of a normal distribution, *λ* is the spatial error coefficient of the vector of the interpreted variable of the *I* × *j* cross-section, ρ and δ measure the spatial lag effects of dependent and independent variables of neighboring geographic units, respectively, $\gamma_{t}$ and $\varepsilon_{it}$ are the random error values of the time and spatial effects, correspondingly. The general SPM can be transformed into three common sub-models:

(1) If ρ = 0 and δ = 0, then the SPM is simplified into the spatial error model (SEM). This model mainly focuses on the spatial autocorrelation between residual terms of explanatory variables of region *i*. The larger the value of *λ*, the greater the spatial influence of the error term on the dependent variable.

(2) If *λ* = 0 and δ = 0, then the SPM is simplified into the spatial lag model (SLM). The model tests the spatial correlation coefficient *ρ* between independent variables of region *i* and its adjacent regions. 

(3) If *λ* = 0, then the SPM is simplified to the spatial Durbin model (SDM). The model considers the spatial correlation of both independent and dependent variables of adjacent regions.

## 3. Results

### 3.1. Description Analysis

**Figure 4:**
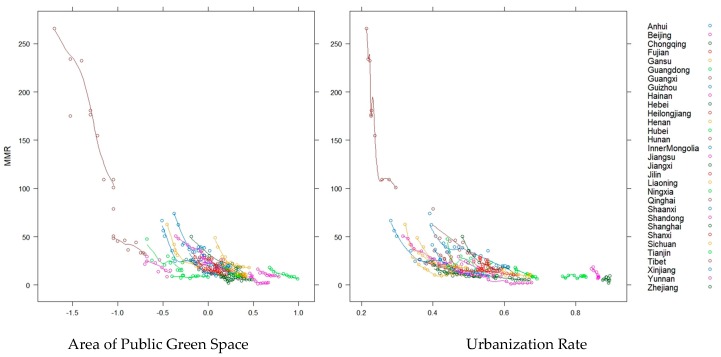
Scatter plot of urbanization rate and area of public green space with maternal mortality ratio (MMR).

Table 1 provides basic descriptive statistics on several characteristic variables, which also indicate significant inequalities in maternal health, socioeconomic development, and green spaces in all provinces. Figure 4 further shows that with the rapid development of urbanization, economic and health care enhancements have greatly improved maternal health. 

**Figure 5:**
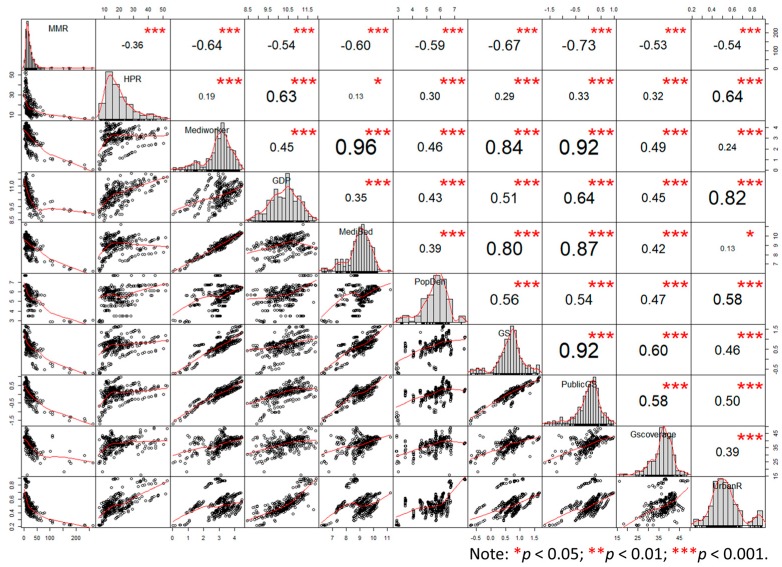
Correlation analysis of all variables by R. Note: MMR (maternal mortality ratio), HPR (high-risk parturient ratio), Mediworker (medical workers), GDP (gross domestic product), PopDen (population density), GS (green space), Public GS (public green space), GS coverage (green space coverage ratio),urban R (urbanization rate). The distribution of each variable is shown on the diagonal, and the bivariate scatter plots with a fitted line are on the left side of the diagonal, while the value of the correlation plus the significance level as stars is on the right side.

Based on an overall correlation of all variables in Figure 5, MMR shows a high correlation with all other variables, which indicates that the enhancement of green spaces, socioeconomic conditions, and medical levels are related to the decline of the MMR in China. In terms of green space variables, public GS gives the highest coefficient, then GS. It is also noteworthy that HPR displays a completely opposite feature, wherein the high connection to GDP might imply that higher HPR tend to appear in regions with better economic conditions, leading to lower MMR. Due to potential collinearity between Mediworker and MediBed, this study deleted the variable of Medibed in further analysis.

### 3.2. Global Moran’s I Statistics

**Figure 6:**
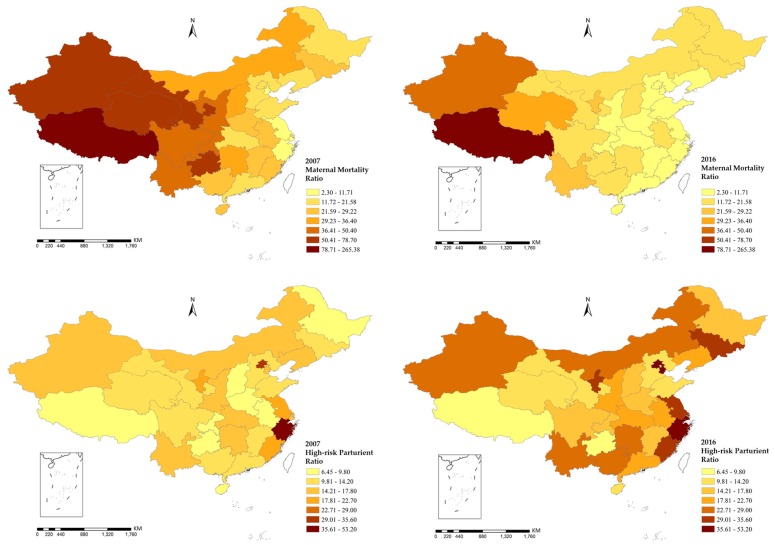
Geographical distribution of MMR and HPR.

Figure 6 illustrates the spatial distribution of MMR and HPR of 31 provinces of China in 2007 and 2016, indicating that maternal mortality shows a clear pattern of spatial autocorrelation. Such spatial features can also be verified by the global Moran’s I statistic in Table 2. All the results of MMR are significant at the *p* = 0.001 level with a slight decline from 2007 to 2016. However, no significant spatial autocorrelation has been found for the variable of HPR.

### 3.3. Panel Data Model

R with a plm package is utilized to apply the PDM, which reported the regression results of pooled OLS, fixed effects, and random effects with high statistical significance (Table 3). 

In the pooled OLS model, the economic indicator of GDP shows a positive correlation to the increase of MMR, while the variables of urbanization rate and population density are negative. GS also implies a similar opposite correlation when compared to Public GS and GS coverage. As for the fixed effect model, HPR and urban R are associated with MMR, wherein urban R plays a dominant role in MMR increasing. All three kinds of green space show potential connections to MMR decrease, while public GS and MediWorker also impose a positive effect on the decline of MMR. The random effect model also indicates a negative relation between MMR and public GS and GS coverage.

An *F* test and Hausman test were applied to further confirm which model is more suitable. Both results show a statistic significance which indicates that the fixed effect (FE) model leads to a more reasonable result. According to FE model, every one percent increase of public green space will decrease about 60 deaths per 10,000 live births.

### 3.4. Spatial Panel Model

A further spatial panel model was applied with R and the splm packages, and all the three kinds of model were also reported (Table 4). In Table 4, the spatial effect coefficient *ρ* (*p* > 0.05) estimated by both SDM and SLM have not shown a statistical significance, neither has the coefficient *λ* (*p* > 0.05) estimated by the spatial error model. Such results indicate that there is no spatial dependence in MMR and residual errors between different provinces. 

Except for the neglectable spatial effect, both SLM and SEM show a similar result as the FE model in the former PDMs, which is that adequate health care conditions and green spaces could prohibit the increase of MMR, while an increase in urbanization rate would propel it.

The coefficient *δ* (*p* < 0.001) in SDM with high statistical significance indicates that the explanatory variables of neighbor provinces, green space coverage, and urbanization rate would impose noticeable effects on MMR in each province.

## 4. Discussion 

The initial purpose of this study was to validate the associations between maternal health and green spaces in the context of rapid urbanization through longitudinal data. The findings indicate that over time, all kinds of green space would benefit the health of pregnant women by lowering MMR, which is consistent with previous studies that have shown how green space might improve maternal health [21,22,23,24,25,26,27,28]. However, there are indeed different influences amongst various kinds of green spaces, wherein public green spaces in particular may play a key role in ensuring maternal health. Such a result could support the positive correlation between parks and pregnancy proposed by previous literature [45,46].

This result can be explained by the fact that different kinds of green spaces represent different mechanisms for impacting maternal health, in which public green spaces mainly show the capacity for physical activities, while green space coverage focuses on the overall environmental quality of a built-up area. The potential for physical activities afforded by public green spaces may be most beneficial to maternal health in both physical and mental conditions. 

In terms of spatial autocorrelation, only green space coverage is slightly related to the reduction of MMR. A possible reason may be that green space coverage of nearby cities would affect environmental quality, as in a previous study that has supported the idea that green space could improve air quality [47] and affect public health in a regional geographic scale.

What is surprising is that the urbanization rate is positively connected with MMR, in short, higher urbanization rates lead to higher MMR, though medical care of a higher level could help reduce MMR during urbanization. This result confirms the negative correlation between urbanization and public health in many studies [48], which indicates the drawbacks of urbanization, such as inadequate and inequality in health care services, the mental health problems of long-distance commute, air and water pollution, housing, poverty, etc. [49]. Meanwhile, Figure 5 shows a strong connection between HPR and urbanization, which matches those observed in early studies [8]. As HPR is positively correlated with MMR in Table 4, it can be concluded that HPR remains an important factor for MMR. Similarly, the unobserved connection between GDP and MMR suggests that higher economic level does not necessarily mean better maternal health.

Certainly, the overall trend of MMR decline could not be neglected, which has been reported in most relevant studies in China [5,6]. The results also show that MMR in China has a significant spatial clustering. According to Table 1, the global Moran’s I of MMR decreased gradually from 2007 to 2016, indicating that the spatial agglomeration of MMR is decreasing. It also means that the Chinese government is already trying to balance regional gaps through public policies and strategies, such as “Western Development”, and “the Rise of Mid-China”, which have changed the polarization of MMR.

## 5. Conclusions 

Generally, the present results suggest a strong tie between public green spaces and maternal health, but also hint at the potential threat to maternal health prompted by the contemporary urbanization process. By 2030, more than 60% of the global population will live in cities [50,51], meaning that the construction of green spaces will be an important part of healthy infrastructure for urban residents. Although urban green spaces have been associated with improving both the physical and mental health of urban residents [52,53], there is huge inequality in the accessibility and construction of green spaces. Green spaces should be taken as an effective measure to alleviate certain health challenges for policymakers.

Compared with traditional PDM, SPM can further explore the spatial connection between independent and explanatory variables, providing robust and profound analysis for the exploration of correlation with longitudinal data. The main weakness of the current study was using provincial geographic units as individuals, which limits the accuracy of the experiment. An additional stratified analysis of urban-rural and high-low socioeconomic status may need to be further explored to provide more information on geographic disparity in maternal health. Other possible factors affecting maternal health, such as housing conditions, air pollution, etc., should also be added. 

## Figures and Tables

**Table 1:** Descriptive statistics of variables.

	2007	2008	2009	2010	2011	2012	2013	2014	2015	2016
	MMR (1 death/100,000 livebirth)									
Min.	7.86	6.57	5.20	3.60	1.20	1.40	1.90	1.90	1.90	2.30
Mean	39.87	32.11	28.84	25.81	22.15	20.66	19.78	17.72	17.72	16.32
Max.	265.38	233.96	232.20	174.80	180.70	176.10	154.50	108.90	108.90	100.90
	HPR (1 death/100,000 livebirth)									
Min.	6.45	6.47	6.50	7.20	5.90	7.60	8.70	8.50	8.50	8.70
Mean	14.28	16.20	17.26	18.17	18.61	19.43	20.77	22.39	22.39	24.68
Max.	38.30	41.19	41.90	42.60	42.50	44.00	46.40	46.90	46.90	53.20
	Area of Green Space (100 km^2^)									
Min.	0.20	0.20	0.22	0.21	0.29	0.34	0.36	0.42	0.53	0.62
Mean	5.56	5.69	6.43	6.89	7.24	7.64	7.83	8.15	8.61	8.99
Max.	27.47	37.70	40.16	42.04	41.06	40.17	41.20	42.19	43.84	45.27
	Area of Public Green Space (100 km^2^)									
Min.	0.02	0.03	0.04	0.03	0.05	0.05	0.06	0.07	0.09	0.09
Mean	1.09	1.18	1.30	1.42	1.56	1.67	1.77	1.86	1.98	2.11
Max.	4.76	5.02	5.32	5.85	6.80	7.40	7.89	8.32	8.96	9.75
	Green Space Coverage Ratio (%)									
Min.	24.10	25.10	27.30	16.00	24.10	30.00	18.10	30.80	29.80	31.10
Mean	34.18	35.32	36.70	36.33	37.75	38.46	38.13	39.34	39.11	39.17
Max.	42.80	42.60	47.70	46.60	46.80	46.20	47.10	49.10	48.40	48.40
	Urbanization rate (%)									
Min.	0.21	0.22	0.22	0.23	0.23	0.23	0.24	0.26	0.28	0.30
Mean	0.47	0.48	0.49	0.51	0.52	0.53	0.54	0.56	0.57	0.58
Max.	0.89	0.89	0.89	0.89	0.89	0.89	0.90	0.90	0.88	0.88
	Health worker (10,000 persons)									
Min.	1.02	1.17	1.60	1.67	2.22	2.16	2.47	2.65	2.91	2.92
Mean	19.06	19.90	25.10	26.44	27.76	29.38	31.55	32.98	34.46	36.01
Max.	45.21	47.98	60.21	64.59	68.96	73.89	81.93	83.85	85.57	87.41

Note: 310 observation from 31 provinces in 10 years.

**Table 2:** Global Moran’s I of MMR and HPR from 2007 to 2016.

Year	Maternal Mortality Ratio (MMR)			High-Risk Parturient Ratio (HPR)		
	Moran’s I	*p*	Z	Moran’s I	*p*	Z
2007	0.162	<0.001	3.552	−0.064	0.678	−0.415
2010	0.130	<0.001	3.166	0.027	0.939	0.007
2013	0.122	<0.001	3.23	0.012	0.56	0.584
2016	0.114	<0.001	3.209	0.032	0.402	0.838

Note: *p* stands for probability and Z stands for standard deviation. The confidence is 90% when Z < −1.65 or Z > 1.65 and *p* < 0.1, the confidence is 95% when Z < −1.96 or Z > 1.96 and *p* < 0.05, and the confidence is 99% when Z < −2.58 or Z > 2.58 and *p* < 0.01.

**Table 3:** Panel model regression results of MMR.

Variables	Pooled OLS	Fixed Effects	Random Effects
HPR	−0.145	0.828 ***	0.516 **
Mediworker	−5.689	−37.711 ***	−10.38
GDP	9.020 *	0.929	0.373
PopDen	−5.603 **	5.733	0.384
GS	16.021 *	−16.624 *	−4.442
Public GS	−42.580 ***	−60.312 ***	−46.133 ***
Gscoverage	−0.774 *	−0.418 *	−0.747 ***
Urban R	−66.523 **	184.193 ***	−2.629
R2	0.618	0.579	0.502
Adjusted R2	0.608	0.52	0.489
*F* Statistic	60.984 *** (df = 8; 301)	46.595 *** (df = 8; 271)	303.620 ***

Note: * *p* < 0.05; ** *p* < 0.01; *** *p* < 0.001.

**Table 4:** Panel model regression results of MMR.

Variables	SDM		SLM		SEM	
	Coef.	*p*	Coef.	*p*	Coef.	*p*
HPR	0.087381	0.645	0.82245	<0.001 ***	0.82801	<0.001 ***
Mediworker	−10.8043	0.024 *	−37.9481	<0.001 ***	−37.4303	<0.001 ***
GDP	−1.91947	0.201	0.886	0.697	0.9084	0.68903
PopDen	−2.6407	0.333	5.71223	0.179	5.66942	0.18136
GS	−2.75892	0.688	−16.841	<0.05 *	−16.891	<0.05 *
Public GS	−10.1467	0.362	−60.0729	<0.001 ***	−60.4161	<0.001 ***
Gscoverage	−0.26285	0.021 *	−0.42238	<0.05 *	−0.42398	<0.05 *
Urban R	−73.6624	0.004 **	183.3074	<0.001 ***	184.0643	<0.001 ***
δ	0.955045	<0.001 ***				
ρ	0.065648	0.3925	−0.016712	0.8125		
λ					−0.016370	0.8378

Note: * *p* < 0.05; ** *p* < 0.01; *** *p* < 0.001. SDM (spatial Durbin model), SLM (spatial lag model), SEM (spatial error model).
